# Quality‐adjusted life years in the presence and absence of organized mammographic screening using data from BreastScreen Norway

**DOI:** 10.1002/ijc.70272

**Published:** 2025-12-12

**Authors:** Rick Groeneweg, Nicolien T. van Ravesteyn, Lindy M. Kregting, Giske Ursin, Solveig Hofvind, Nataliia Moshina

**Affiliations:** ^1^ Department of Public Health Erasmus MC, University Medical Center Rotterdam Rotterdam the Netherlands; ^2^ IQ Health Science Department Radboud University Medical Center Nijmegen the Netherlands; ^3^ The Cancer Registry of Norway Norwegian Institute of Public Health Oslo Norway; ^4^ Department of Nutrition, Institute of Basic Medical Sciences University of Oslo Oslo Norway; ^5^ Department of Preventive Medicine University of Southern California Los Angeles California USA; ^6^ The Cancer Registry of Norway, Department of Screening Programs Norwegian Institute of Public Health Oslo Norway; ^7^ Department of Health and Care Sciences UiT The Arctic University of Norway Tromsø Norway

**Keywords:** breast cancer, mammographic screening, quality‐adjusted life years

## Abstract

Benefits and harms of breast cancer (BC) screening with mammography have been debated and, although most studies reported positive effects, some studies found a negative effect in terms of net quality‐adjusted life years (QALYs). We aimed to estimate net QALYs associated with biennial mammographic screening for women aged 50–69 years offered to 100,000 women followed until age 85, using various assumptions on BC mortality reduction, overdiagnosis and mortality transfer (the extent to which a reduction in BC mortality results in a reduction in all‐cause mortality). Individual‐level data from women invited to BreastScreen Norway during 1996–2020 were used to perform the calculations. The three baseline scenarios included (1) Model Microsimulation Screening Analysis (MISCAN): MISCAN prediction for mortality reduction and overdiagnosis proportion; (2) Model A: 40% BC mortality reduction and 15% overdiagnosis; and (3) Model B: 20% BC mortality reduction and 50% overdiagnosis. For all scenarios, an 80% mortality transfer was assumed. An online tool was developed to illustrate the impact of alternative assumptions. Biennial organized mammographic screening for women aged 50–69 years who were followed until the age of 85 years was associated with 6819, 7444 and 2446 net QALYs gained per 100,000 women for Model MISCAN, A and B, respectively. Assumptions on BC mortality reduction exhibited the largest impact on net QALYs. To conclude, even when assuming a high overdiagnosis proportion and low BC mortality reduction, net QALYs remained positive, reinforcing the value of offering BC screening with mammography to Norwegian women and showing its potential to improve health outcomes.

## What's New?

Although mammographic screening can reduce breast cancer mortality and extend life, some studies have found a negative impact on net quality‐adjusted life years (QALYs). Here, the authors estimated net QALYs associated with biennial mammographic screening for women aged 50–69 years in Norway under various scenarios. The screening program resulted in a gain of 2446–7444 net QALYs per 100,000 women when followed up until 85 years of age. Even when assuming a high overdiagnosis proportion and low breast cancer mortality reduction, net QALYs remained positive, reinforcing the value of offering mammographic screening and its potential to improve health outcomes.

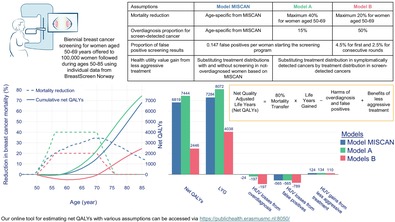

**Abbreviations**
BC: breast cancer
BCT: breast conserving treatment
DCIS: ductal carcinoma in situ
HT: hormonal therapy
HUV: health utility value
LYG: life‐years gained
MISCAN: Microsimulation Screening Analysis
OdP: overdiagnosis proportion
QALY: quality‐adjusted life year

## 1 BACKGROUND

In 2020, breast cancer (BC) was the most common cancer and leading cause of cancer death among women worldwide.^1^ Over the last decades, BC mortality was observed to be reduced due to improved treatment and implementation of organized screening programs.^2^, ^3^, ^4^ Asymptomatic screen‐detected BC is associated with more favorable prognostic and predictive histopathologic tumor characteristics than symptomatic BC.^5^ Therefore, women with symptomatic cancer receive more aggressive treatment than women with screen‐detected cancer, which might influence their quality of life.^5^, ^6^, ^7^ Besides the positive effects, participation in mammographic screening can also negatively affect a woman's quality of life due to anxiety related to false‐positive screening results and overdiagnosis and overtreatment of low‐proliferating tumors.^8^, ^9^

The effects of mammographic screening can be investigated by comparing the effects of benefits, including mortality reduction and life‐years gained (LYG), to the harms, including false‐positive screening results and overdiagnosis.^9^, ^10^, ^11^ These effects can be measured using quality‐adjusted life years (QALYs).^12^ QALYs combine the length and the quality of life, indicating the person's ability to perform usual activities without pain and mental disturbance.^12^ If the quality of life is represented by a health utility value (HUV) measured on a scale where 0 represents “death” and 1 “perfect health,” the number of QALYs is estimated by multiplying the expected length of life by the expected quality of life shown in HUV.^12^ Modelling studies on the effect of mammographic screening programs on QALYs have reported controversial findings, including both substantial and low net benefits or even net QALYs lost.^11^, ^13^, ^14^, ^15^, ^16^ Possible side effects and long‐term effects of BC treatment have changed over time and adjusting for these effects might influence the amount of net QALYs both positively, if the treatment extent is reduced and negatively, if overtreatment occurs.^17^ Moreover, the harms from overdiagnosis and subsequent overtreatment included in the net QALYs calculations of several previous studies were based solely on the pre‐screening incidence.^11^, ^14^ In addition, some of the previous studies assumed that the effect of BC mortality reduction did not extend for more than 5 years after the screening period finished, as the studies lacked sufficient follow‐up for women after screening ages.^11^, ^14^, ^15^, ^18^ Results from recent studies and Microsimulation Screening Analysis (MISCAN) showed that mortality reduction due to mammographic screening greatly extended into older ages.^19^, ^20^, ^21^, ^22^

In Norway, reporting of the information pertaining to cancer cases to the Cancer Registry of Norway has been mandatory since 1953.^23^ Further, data related to the screening program, BreastScreen Norway, have been systematically collected since 1996, while detailed data on breast cancer treatment have been available since 2013^24^, ^25^ and self‐reported treatment information was collected for the period 2006–2017.^26^ This study aimed to use these individual‐level data to estimate QALYs for scenarios including an organized mammographic screening program as compared to no available organized mammographic screening. More specifically, we aimed to perform a study similar to a Norwegian study reporting a loss of net QALYs due to mammographic screening^11^ and elaborate on various assumptions on the BC mortality reduction due to screening, on overdiagnosis and on BC treatment using individual‐level data and MISCAN.

## 2 METHODS

### 2.1 Data, population and input parameters

We modelled two hypothetical groups of 100,000 Norwegian women, from age 50 to the age of 85 (Figure S1, Supporting Information). One group was invited to mammographic screening; the other group was not invited.

To model the situation with screening, we used data from BreastScreen Norway, an organized screening program offering women with a Norwegian personal identification number and aged 50–69 years two‐view mammographic screening biennially. The program started in 1996 and became nationwide in 2005. During the first 25 years, the annual attendance rate was 75% among those invited (Table 1). The recall rate among the screened women was about 3% and the rate of screen‐detected BC was 0.6%.^24^ Data from BreastScreen Norway included information on women invited from the start of the program (1996) until the year 2020. BC was defined as ductal carcinoma in situ (DCIS) or invasive BC. Screen‐detected cancer was BC diagnosed as a result of a positive screening examination. Interval cancer was BC diagnosed 0–24 months after a negative screening examination or 6–24 months after a false‐positive screening result.^24^ Symptomatic cancer was BC diagnosed among women invited to the screening program, but who either never attended or last attended more than 2 years prior to their diagnosis. Aggregated data on the distribution of different treatment regimens, histological type, and detection mode (screen‐detected and symptomatically detected cancers) were obtained from the Cancer Registry of Norway, Norwegian Institute of Public Health.^26^, ^30^

**TABLE 1:** The main assumptions for the calculations.

Input parameters	Values	Application
Attendance	75%^24^	Calculating number of false positives
False‐positive screening results	Method 1: 4.5% in the first screening round, and 2.5% in consecutive screening rounds.^11^, ^27^ Method 2: a cumulative risk of 0.147^a^ false‐positive results per woman	Calculating number of false positives
Health utility value (HUV) loss from a false‐positive result	For the first month −0.025, and then −0.0125 for 3 years after the false‐positive result^8^	Calculating the harm from false positives
Baseline all‐cause mortality rate 2017^11^	The 1940 female Norwegian birth cohort (up to 2017, and then age‐specific rates in 2017 for the age groups 78–85 years)	Calculating number of women alive by age
Baseline incidence rates of invasive breast cancer per 100,000 women‐years (wy), 1980–1985^28^	120/100,000 wy (50–54 years) 133/100,000 wy (55–59 years) 163/100,000 wy (60–64 years) 197/100,000 wy (65–69 years) 223/100,000 wy (70–74 years) 256/100,000 wy (75–79 years) 265/100,000 wy (80–85 years)	Calculating number of breast cancer cases not overdiagnosed
Baseline breast cancer mortality rates per 100,000 women‐years (wy) (1980–1985)^28^ (NORDCAN)	40/100000 wy (50–54 years) 56/100000 wy (55–59 years) 73/100000 wy (60–64 years) 76/100000 wy (65–69 years) 102/100000 wy (70–74 years) 115/100000 wy (75–79 years) 142/100000 wy (80–85 years)	Calculating number of breast cancer deaths
Age frame during the study period	50–85 years (in the baseline scenarios); 50–95 years (Figure S10)	Calculating number of life years gained
Estimated HUV losses	EQ‐5D‐5L^b^, ^11^, ^12^	Calculating quality‐adjusted life years

*Note*: EQ‐5D‐5L is a EuroQol Group (EQ) instrument for measuring health related quality of life consisting of five dimensions (5D, mobility, self‐care, usual activities, pain/discomfort and anxiety/depression) with five levels of severity (5L, no problems, slight problems, moderate problems, severe problems and extreme problems).^29^

^a^ The calculation is based on individual data from BreastScreen Norway.

^b^ The HUV losses were per 10 years, as a summation of the yearly HUV, based on alternative, equity‐weighted utilities presented in a previous study from Norway^11^ (table S1 in their publication).

For BC incidence and mortality, we used the average population rates in the period 1980–1985 (before start‐up of BreastScreen Norway in 1996).^28^, ^31^ The number of new BC cases per year was calculated as the BC incidence multiplied by the number of women alive who have never had a BC diagnosis. Using population BC mortality rates, we calculated the number of women at risk for BC and BC deaths in the groups of women invited and not invited to an organized breast cancer screening program. Women could have died from BC or from other causes. The other‐cause mortality was calculated as the difference between all‐cause mortality and BC mortality.^28^, ^32^ The data on all‐cause mortality were obtained from the supporting information used in Zahl et al.^11^ Women who were diagnosed with BC but did not die from the disease (including women assumed to be saved by screening and the overdiagnosed women), died at the rate of the other‐cause mortality. In our analyses, the upper age limit was 85 years. As previous studies showed a time and thus an age‐specific effect of screening on BC mortality,^19^, ^33^ supplementary analyses with the upper age limit varying from 80 to 95 years were performed to assess the impact of follow‐up time on the outcomes (BC mortality reduction and net QALYs).

### 2.2 Benefits of breast cancer screening

#### 2.2.1 Breast cancer mortality, life‐years gained, and mortality transfer

**FIGURE 1:**
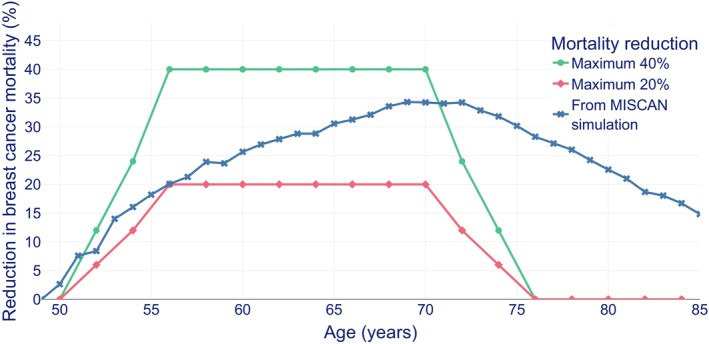
Reduction in breast cancer mortality by age as used in previous studies; green: a maximum reduction in breast cancer mortality during ages 55–70 of 40%^34^; red: a maximum reduction in breast cancer mortality during ages 55–70 of 20%^36^; dark blue: reduction in breast cancer mortality based on MISCAN simulation.^21^

BC mortality was defined as mortality from BC based on the incidence in the assessed age groups of women.^28^ The number of life years gained (LYG) due to screening was modelled by reducing the BC mortality on a per‐age basis. We used different levels of this BC mortality reduction as a function of age based on literature^11^, ^19^, ^34^, ^35^, ^36^ (Figure 1). Two shapes of BC mortality reduction were modelled: (1) the trapezium shape with 0 reduction at age 50, gradually rising to a set maximum from age 56 to 71, and gradually declining to 0 at age 75; (2) the MISCAN shape based on computing the BC mortality reduction from running MISCAN with and without a biennial mammographic screening program from age 50 to 70. The trapezium shape was modelled for consistency with a previous study on QALY effects of BC screening from Norway.^11^

The maximum BC mortality reduction at a certain age range was not considered equal to the total BC mortality reduction over the age range of 50–85 years, for instance, the BC mortality reduction labeled maximum 40% was equal to 40% solely for ages 55–70, while the total BC mortality reduction was 19.5% when expressed as a percentage over ages 50–85 (Figure S2). The total BC mortality reduction was calculated by dividing the total number of BC deaths prevented due to invitation to screening, by the total number of BC deaths in the absence of screening. The total number of BC deaths prevented depended only on the BC mortality reduction and input parameters shown in Table 1.

The number of LYG was influenced by the extent to which a reduction in BC mortality translated into a reduction in all‐cause mortality.^37^ More precisely, LYG from reducing BC mortality, transferred to the LYG from a reduced all‐cause mortality by a factor that was referred to as mortality transfer.^11^, ^37^ Mortality transfer was therefore an assumed proportion of LYG for various estimates of BC mortality reduction.^11^, ^34^, ^35^, ^36^ This proportion could be lower than 100% due to various reasons; for example, as a result of deaths due to heart disease and lung cancer in association with radiation therapy of early‐stage breast cancer.^11^, ^37^ Based on previous studies, mortality transfers of 50%, 80% and 100% were included in our analyses.^11^, ^37^

### 2.3 Harms of breast cancer screening

#### 2.3.1 False‐positive screening results

The rate of false‐positive screening results was defined as the percentage of screening examinations resulting in a recall but no BC diagnosis.^27^ Calculation of the number of false‐positive screening results was performed using two methods (Table 1). First, the number of false positives was considered a fixed percentage of the first screening examinations (4.5%), and a fixed percentage of consecutive screening examinations (2.5%) assuming an attendance rate of 75% among all the women in the target group of the screening program, consistent with a previous study from Norway.^27^ Second, using the data from BreastScreen Norway, we estimated the average cumulative number of false positives per woman invited to screening at the age of 50. The number of false positives in the age range 50–69 years divided by the number of women who started screening at the age of 50 years was 0.147. We estimated that 14,700 false‐positive screening examinations would thus occur in the group of 100,000 women.

#### 2.3.2 Overdiagnosis

Overdiagnosis was defined as receiving a BC diagnosis that would not have been received in the absence of screening.^9^, ^38^ The harm of overdiagnosis was estimated as the harm associated with the treatment of overdiagnosed women. To model the number of overdiagnosed women per year, we assumed the incidence of not‐overdiagnosed BC cases to be equal to the incidence from 1980 to 1985 (Table 1). Further, we added overdiagnosed cases to this incidence, constraining the number of overdiagnosed cases to be proportional to the number of screen‐detected cancers in each age group (50–54, 55–69, 60–64 and 65–69 years) (see Overdiagnosis calculation section in Data S1). This resulted in a model where the non‐overdiagnosed incidence remained equal to the incidence before screening, and a fixed proportion (overdiagnosis proportion, OdP) of the screen‐detected cancer cases was overdiagnosed (Figure S3).

In addition, we made two alternative assumptions about what the number of overdiagnosed women was proportional to (see Data S1 for more details). First, the number of overdiagnosed cases was assumed to be proportional to the incidence from the period before screening started (1980–1985) without considering the number of screen‐detected cancers, as performed in a previous study from Norway^11^ (Figure S4). Second, the cumulative number of overdiagnosed cases was estimated based on the various percentages of screen‐detected DCIS and invasive cancer cases being overdiagnosed (Figure S5).

### 2.4 Modelling treatment

Side and long‐term effects of BC treatment have been reported to be influenced by detection mode.^11^, ^14^, ^15^, ^34^ First, screening has been shown to detect BC at an earlier stage with no symptoms and thus results in less aggressive treatment compared to the treatment of women with symptomatic BC.^5^ Second, treatment of overdiagnosed women results in harms associated with reduced quality of life in these women.^11^, ^14^

**FIGURE 2:**
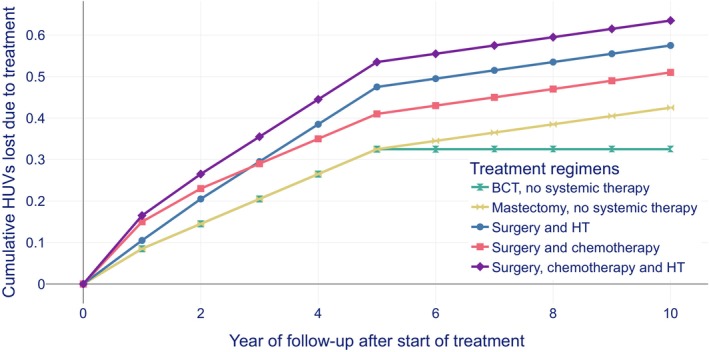
Assumed harms in health utility values (HUVs) lost due to breast cancer treatment regimens during a 10‐year follow‐up period. BCT refers to breast conserving treatment. Surgery refers to BCT or mastectomy with or without radiation therapy. HT refers to hormonal therapy. Systemic therapy refers to chemotherapy and/or HT.

While modelling BC treatment, five treatment options were included in our analyses: breast conserving treatment (BCT), mastectomy, radiation therapy, hormonal therapy (HT) and chemotherapy.^11^, ^26^, ^30^ We defined a treatment regimen as the combination of different treatment options a woman received (e.g., mastectomy with radiation therapy and HT). Surgery was referred to as BCT or mastectomy with or without radiation therapy. We identified five types of treatment regimens for screen‐detected BC cases: (1) BCT with or without radiation therapy; (2) mastectomy with or without radiation therapy; (3) surgery and HT; (4) surgery and chemotherapy; and (5) surgery, HT and chemotherapy. We assumed that each treatment regimen was associated with HUV loss (see health utility values [HUVs] and net quality‐adjusted life years [QALYs] section for details). HUV losses for the aforementioned treatment regimens were estimated per year (Figure 2) and per 10 years, as a summation of the yearly HUV (Table 2 and Figure 2), based on alternative, equity weighted utilities presented in a previous study from Norway^11^ (Table S1 in their publication).

**TABLE 2:** Treatment regimens for women assumed overdiagnosed with cumulative harm estimated in health utility value (HUV) loss during a 10‐year follow‐up period.

Treatment regimen^a^	Treatment distribution in overdiagnosed women (%; range used in sensitivity analyses)	Cumulative harm during 10‐year follow‐up per woman (total HUV loss)^11^
Breast conserving treatment (BCT) with or without radiation therapy	28.8% (20%–40%)	0.325
Mastectomy with or without radiation therapy	19.2% (10%–30%)	0.425
Surgery (BCT or mastectomy with or without radiation therapy) and hormonal therapy	32% (24%–40%)	0.575
Surgery and chemotherapy	12% (0%–24%)	0.510
Surgery, hormonal therapy and chemotherapy	8% (0%–16%)	0.635

^a^ Based on modified treatment modalities and alternative, equity‐weighted utilities presented in table S1 in Zahl et al.^11^

#### 2.4.1 Modelling benefits of less aggressive treatment

While modelling treatment, we took into account changes in treatment intensity due to cancers being diagnosed at an earlier stage compared to the situation when cancers would have been diagnosed without screening. Some women with screen‐detected BC would have been diagnosed with a tumor in a later stage and received a different treatment regimen in the absence of a screening program. To estimate the extent of treatment regimens administered, screen‐detected BC cases were presented in five groups: (a) DCIS that would have been diagnosed as DCIS without screening. (b) DCIS that would have been diagnosed as invasive BC without screening. (c) DCIS that would not have been diagnosed without screening. (d) Invasive BC that would have been diagnosed later during a woman's lifetime if she had not been screened. (e) Invasive BC that would not have been diagnosed during a woman's lifetime. We used these five groups to model the received treatment regimen by age. Since the proportion of DCIS for women with symptomatic cancers was not available, we used the proportion of DCIS identified among the interval cancers as a proxy, ranging 5–7%.^24^ The proportion of DCIS among women with screen‐detected cancer was 19% (14–24%) based on the individual data from BreastScreen Norway. For the invited group, the proportions of DCIS and invasive BCs were obtained from the individual age‐specific data, while the numbers could differ depending on how many women were diagnosed at different age groups given the incidence data from Table 1.

We assumed that screen‐detected BC Groups a, b and d had benefits from a less aggressive treatment. Groups c and e were considered overdiagnosed. Women in these two groups were negatively affected by treatment. To model the change in treatment in Groups a, b and d, we used the difference in the distribution of treatment regimens for screen‐detected and symptomatic BC (DCIS and invasive BC separately). Proportions of women invited to BreastScreen Norway, diagnosed with screen‐detected, symptomatic and interval cancer and who received various treatment regimens based on self‐reported information from responses to the questionnaire on health‐related quality of life were shown in Table S1. These proportions were used to estimate the number of women who could benefit from less aggressive treatment and lower HUV loss due to screening. HUV losses from the change in treatment presented in Figure 2 were used to estimate the number of gained QALYs.

#### 2.4.2 Modelling harms of overtreatment in overdiagnosed women

Based on observed frequencies of the various diagnoses and treatment pathways in Norway (Table 2), the expected total impact of a treatment regimen was estimated as HUV loss during 10 years of follow‐up.^11^, ^26^, ^30^ The harms caused by the five treatment regimens were plotted assuming 10‐year survival (Figure 2).

### 2.5 Health utility values and net quality‐adjusted life years

HUVs were obtained from a previous study on QALYs associated with breast cancer screening in Norway (Table 2).^11^ The HUV loss for women with a false‐positive result was 0.025 for the first month, and 0.0125 for each month during the three following years after the false‐positive result (Table 1).^8^

Net QALYs were calculated using the formula:

NetQALYs=LYG·mortality transfer−HUVlosses+HUVgains,

where HUV losses were due to false‐positive screening results, overdiagnosis, and side and long‐term effects of treatment during the 35 years of follow‐up. HUV gains were the benefits for women who received less aggressive treatment due to screening.

### 2.6 Scenarios

We used various assumptions on BC mortality reduction and overdiagnosis, based on existing literature^11^, ^19^, ^34^, ^36^, ^38^ (Table 1), resulting in three baseline scenarios: Model MISCAN, Model A and Model B (Table 3).

**TABLE 3:** Differences in assumptions for the three baseline scenarios.

Assumptions	Model MISCAN	Model A	Model B
Mortality reduction	Age specific numbers from MISCAN simulation (Figure 1)	Trapezium shape with a maximum of 40%^34^	Trapezium shape with a maximum of 20%^36^
Overdiagnosis proportion for screen‐detected breast cancer (OdP)	Age specific proportion of screen‐detected cases from MISCAN (Figure S6)	15%^38^	50%^11^
Method to calculate number of false positives	From BreastScreen Norway dataset (0.147 false positives per women invited to the screening program)	4.5% in the first screening round and 2.5% in consecutive screening rounds^11^	
Health utility value gain from less aggressive treatment	Substituting the treatment distributions with and without screening in not‐overdiagnosed women from MISCAN (Figure S7 with harms from treatment as in Figure 2)	Substituting the distribution of treatment in symptomatically detected for screen‐detected cancers from treatment distribution data from Norway (with harms from treatment as in Figure 2)	

Model A and Model B were the result of extreme assumptions on breast cancer mortality and overdiagnosis from available literature,^11^, ^19^, ^32^, ^34^, ^37^ while model MISCAN was an alternative with more flexible estimates of mortality for ages 50–85 years, as well as overdiagnosis based on the screening data (Figure S6).^21^ All three scenarios were based on a mortality transfer of 80%.

### 2.7 Statistical analyses

The cumulative LYG, other combined benefits (less aggressive treatment) and harms (false positives and overdiagnosis) of an organized BC screening program, and net QALYs were plotted for the three baseline scenarios (model MISCAN, Model A, and Model B) using combinations of various assumptions presented in Table 3 for women aged 50–85 years in the presence of BC screening. Further, the total net QALYs gained and the number of QALYs associated with the benefit of LYG, harms of overdiagnosis and false positives and the benefit of less aggressive treatment were shown per 100,000 women for the three scenarios with a mortality transfer of 80% for 35 years of follow‐up (50–85 years).

Finally, the sensitivity analyses on the variation of net QALYs gained (or lost) by different levels of mortality transfer, maximum age of follow‐up, BC mortality reduction and OdP were depicted in tornado plots for the three baseline scenarios. The effect of screening on treatment predicted by MISCAN is presented in Figure S7a–d. Sensitivity analyses of the treatment harm for overdiagnosed women, depending on the treatment distribution that overdiagnosed women were modelled to receive, were presented as tornado plots for Model A and B in Figure S8a,b. Estimated numbers of prevented systemic therapies were plotted in Figure S9. An online tool presenting the net QALYs for various multiple assumptions on benefits and harms of BC screening can be accessed via https://publichealth.erasmusmc.nl:8050/.

The models were written and run in Python (version 3.10.11, https://www.python.org/psf/about/), with NumPy (version 1.23.5, https://numpy.org/doc/stable/). Data analyses were performed in the same programming language using Polars (version 0.19.12, https://docs.pola.rs) and Pandas (version 1.4.4, https://pandas.pydata.org/docs/). Figures were generated with Plotly (version 5.9.0, http://plotly.com/python).

## 3 RESULTS

**FIGURE 3:**
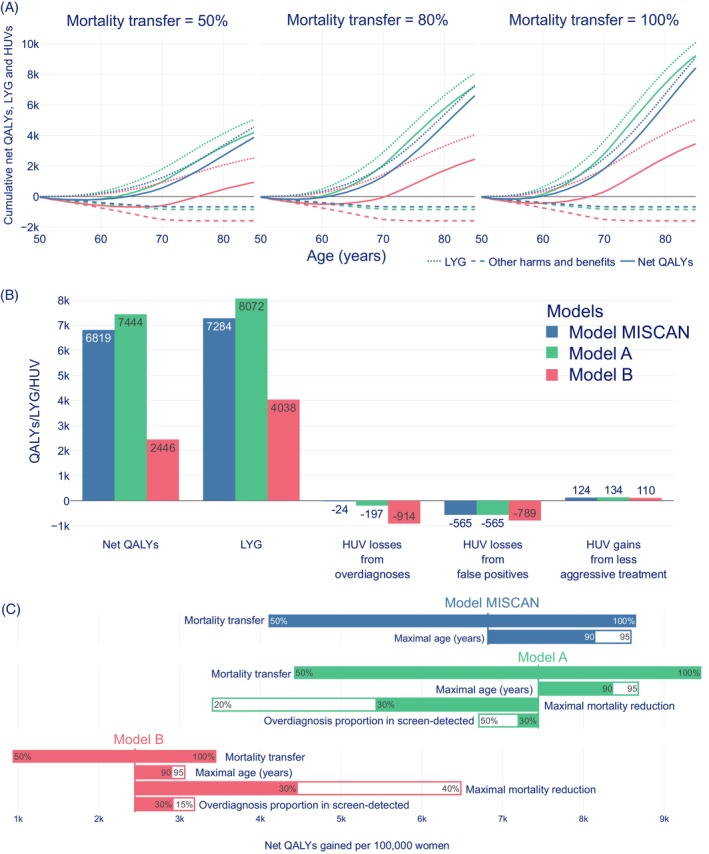
(A) The cumulative quality‐adjusted life years (QALYs), life years gained (LYG) and other harms and benefits for Model Microsimulation Screening Analysis (MISCAN) (blue), Model A (green) and Model B (red) and mortality transfer of 50%, 80% and 100% per 100,000 women aged 50–85 years. (B) Net QALYs gained due to screening, composed from LYG, harm of overdiagnosis, harm of false‐positive results and benefit of less aggressive treatment for Model MISCAN (blue), Model A (green) and Model B (red) and mortality transfer of 80% per 100,000 women aged 50–85 years. (C) A sensitivity analysis for the impact of the variation of mortality transfer, maximal age (100 years), BC mortality reduction and overdiagnosis proportion on net QALYs gained per 100,000 women for Model MISCAN (blue), Model A (green) and Model B (red).

An organized breast cancer screening program inviting women aged 50–69 years biennially was associated with a gain of 6819, 7444 and 2446 net QALYs per 100,000 women for Model MISCAN, Model A and Model B, respectively, using a mortality transfer of 80% and a follow‐up period until 85 years (Figure 3a,b and Table S2). Benefits associated with BC mortality reduction resulted in 7284, 8072 and 4038 LYG for Model MISCAN, Model A and Model B, respectively, while harms related to overdiagnosis resulted in the loss of 24, 197 and 914 QALYs. Benefits in terms of less aggressive treatment resulted in 124, 134 and 110 QALYs gain, respectively. Harms due to false positives led to the loss of 565 QALYs for Model MISCAN and Model A, and 789 QALYs for Model B, respectively.

In the short term, the harms of screening (false positives) are more prominent, while the benefits of screening become apparent later. During the first 8–16 years of follow‐up, the cumulative net QALYs were negative due to the outweighing harms of overdiagnosis and false positives. At different levels of mortality transfer, the ages at which net QALYs became positive varied (Figure 3a). For a mortality transfer of 50%, net QALYs turned positive at the age of 64 years for Model MISCAN, 62 years for Model A and 76 years for Model B. These turning points were observed at the age of 61, 59 and 71 for a mortality transfer of 80%, and at the age of 60, 58 and 68 for a mortality transfer of 100%, with the same model order. The increase in LYG was the main contributor to outweighing benefits over harms resulting in positive net QALYs.

For Model MISCAN, BC screening was estimated to result in a net QALYs gain between 4102 and 8654 for a mortality transfer of 50 and 100%, respectively (Figure 3c). For the same variation of mortality transfer, net QALYs gained varied between 4417 and 9462 for Model A, and between 931 and 3455 for Model B. Increasing the maximum age of follow‐up from 85 to 95 years led to additional 1285, 1824 and 642 QALY gains in Model MISCAN, A and B, respectively. Variations in the OdP led to more modest changes in the number of net QALYs, compared to the impact of mortality reduction.

The effect of less aggressive treatment of the screened women accounted for a modest HUV gain (Figure 3a); however, as presented in a sensitivity analysis, systemic therapy was extensively avoided among not‐overdiagnosed women compared to other scenarios (Figure S7a–d).

Considering QALYs lost, false positives showed a larger impact on net QALYs in Models MISCAN and A compared to Model B, whereas overdiagnosis contributed more to net QALYs in Model B. Figure S8a,b showed the sensitivity analysis of the harms from overdiagnosis in Model A and B relative to the assumed treatment proportions.

When a longer follow‐up until the age of 95 years (instead of 85) was taken into account, the cumulative LYG were 4655, 9310 and 9042 for 20%, 40% and MISCAN mortality reduction, respectively (Figure S10). For all assumptions on BC mortality reduction due to screening, there was a substantial increase in LYG due to extending this follow‐up. For MISCAN the increase was steepest, because BC deaths were still prevented at older ages.

## 4 DISCUSSION

Using information from BreastScreen Norway, 80% mortality transfer and various assumptions on BC mortality reduction, overdiagnosis, false‐positive results and less aggressive treatment due to screening, we found that an organized breast cancer screening program inviting women aged 50–69 years biennially resulted in a gain of 2446–7444 net QALYs per 100,000 women for a follow‐up until 85 years of age.

Mortality transfer and BC mortality reduction had the most pronounced impact on the variation of net QALYs in our study, as LYG was by far the largest component of net QALYs. Model MISCAN showed a larger benefit in terms of net QALYs and LYG at older ages than the models using a maximum BC mortality reduction at ages 50–75 years, because MISCAN incorporated a BC mortality reduction after age 75.

Harms of false positives and overdiagnosis were a relatively minor component of net QALYs. The harms of false positives were larger than the harms of overdiagnosis in Models MISCAN and A. This might be caused by the assumption of a long period (3 years) in which quality of life was reduced due to false‐positive results of screened women.^11^ This finding might also be linked to the use of the HUVs which were not equity weighted as compared to HUVs used in another study from Norway estimating net QALYs.^11^ Equity weighting might reduce the value of health deterioration in association with false‐positive results, as equity weighting is used to adjust for various biases in assessment of the health state, including assigning the minor health problems with a larger health loss resulting into lower HUVs.^11^, ^39^ Furthermore, the methods to estimate the OdP in this study differed from those presented previously for net QALYs calculations, as the previous methods were only based on the total breast cancer incidence before screening started, not accounting for proportions of screen‐detected versus symptomatic (including interval) cancers for women invited to screening.^11^, ^14^ This study also presented overdiagnosis as the proportion of all screen‐detected cancers and various proportions of screen‐detected DCIS and invasive cancers. The estimated impact of overdiagnosis differed extensively between Model MISCAN and Model B (−24 vs. −914 QALYs, respectively). In Model B, a level of 50% overdiagnosis in screen‐detected BC might be unrealistically high,^38^ but a level of 15% in Model A still caused 8 times as much harm (−197 QALYs) as in Model MISCAN. In Model MISCAN, this level was much lower at 3.7% at age 50 and 8.9% at age 68 (Figure S6). Age‐dependency of the proportion of overdiagnosed cases was not considered in Models A and B. Furthermore, the differences in treatment distributions widened the differences in the extent of harms from overdiagnosis. In Model MISCAN, systemic therapy for overdiagnosed women was uncommon (Figure S7d), but in Models A and B, the majority of overdiagnosed women received systemic therapy (Table 2). Hence, the harms of treatment caused per overdiagnosed woman were greater in Models A and B than in Model MISCAN.

Previous studies on net QALYs gained due to mammographic screening performed during the last decade showed various findings, with net QALYs gain ranging from 1513 to 23,100 per 100,000 women,^11^, ^21^, ^40^, ^41^ which might be considered in line with our findings, because net QALYs gained were positive. First, in a modelled situation with annual screening at age 40 and switching to biennial screening from ages 50–69, 70% attendance, 15% mortality reduction with a lifetime follow‐up, about 18% overdiagnosis and a 3% discount rate, 4593 QALYs gain was estimated in a population of 100,000 women in Singapore.^40^ Second, a recent study with data from the Netherlands reported 23,100 QALYs gained per 100,000 women in a situation with biennial screening of women aged 50–74 years and 100% attendance.^21^ Third, another study from the Netherlands estimated 13,280 QALYs gained per 100,000 women screened biennially ages 50–69 assuming 80% attendance and 25% mortality reduction.^41^ Lastly, a previous Norwegian study assuming a maximum BC mortality reduction of 20%, mortality transfer of 80% and overdiagnosis of 20% with screening attendance and age at invitation similar to our study, estimated a 1513 net QALYs gain.^11^

Comparing our findings to the results from screening programs for other cancer sites, we also found large ranges in net QALYs estimates. Previous investigations of QALYs gained per 100,000 persons for colorectal cancer screening as biennial fecal immunochemical test for ages 54/55–74/75 years showed a variation from 17,863 QALYs gained in Hong Kong^42^ to 21,500 QALYs gained in Australia.^43^ Further, QALYs gained per 100,000 persons for lung cancer screening as annual low‐dose computer tomography for ages 40/55–64/74 were 5474 in the United States^44^ and 5970 in Denmark.^45^ Our results might be seen in agreement with the findings on QALYs gained for lung cancer screening, while QALYs gained in association with colorectal cancer screening were much higher. The considerable variation in QALYs gained for different screening services was associated with differences in methods of calculation, eligible population, screening modalities and participation rates.^42^, ^43^, ^44^, ^45^ Therefore, direct comparisons should be made with caution.

### 4.1 Strengths and limitations

A major strength of this study is the individual‐level data on all women screened in BreastScreen Norway and data on treatment distribution. Furthermore, the study incorporated possible side effects and long‐term consequences of BC treatment both in terms of the harms from overdiagnosis and subsequent overtreatment and in terms of benefits of less aggressive treatment for those who were diagnosed at an early stage. Overdiagnosis was assessed using data from the screened population supplemented with multiple assumptions. Finally, the MISCAN model, validated on the data from screening programs similar to BreastScreen Norway,^20^, ^22^ was applied to calculate the mortality reduction and number of overdiagnosed cases due to BC screening.

However, several limitations should be mentioned. Some studies questioned the extent to which the BC mortality reduction transferred to all‐cause mortality reduction,^11^, ^46^, ^47^ while other studies implicitly assumed 100%.^21^, ^48^ The choice of 80% mortality transfer was related to our assumption that BC mortality reduction was balanced by mortality increase due to heart disease and lung cancer for women with early‐stage BC who received radiation therapy.^49^ Further, no discounting factor was added in this study, as we aimed at assessing the net QALYs instead of the cost‐effectiveness. Discounting the number of QALYs gained would take into account women's preferences to receive health benefits now rather than in the future.^15^, ^20^ The assumption that OdP was linearly related to the number of screen‐detected BC cases and based on the incidence in the pre‐screening period used for Models A and B might be considered a limitation, as in reality, the OdP will vary with age and histologic type^50^ and the increased incidence during the last decades will be in part attributable to changes in BC risk factors.^51^ Therefore, a relatively simple model for overdiagnosis calculation could have led to a wide range in net QALYs due to various assumptions. More sophisticated models, such as MISCAN, and more detailed data, such as stage by detection mode and OdP by age, are needed for validation and ruling out unrealistic assumptions. Even though the OdP assumptions in Models A and B most likely overestimated the number of overdiagnosed cases, the results still showed a net QALY gain. The MISCAN model was not calibrated for BreastScreen Norway, which might have resulted in both under‐ and overestimation of BC mortality reduction. The treatment distribution that overdiagnosed women received, could not be measured directly. For Models A and B, we adopted assumptions from a previous study from Norway,^11^ but these differed greatly from the results from the MISCAN simulation. This led to a wide range of estimates of the harms from overdiagnosis.^9^, ^11^, ^14^ The QALYs were not adjusted for age and did not account for poorer general health for older women compared to younger women, which might have increased the net QALYs estimate, specifically in model MISCAN.^39^ False‐negative screening results were not considered harm in this study as we assumed that screening was a service that was not designed to detect all BCs. False reassurance could be assumed as harm, but the magnitude of this harm has not been shown.^52^ The level of opportunistic screening and possible associated mortality reduction were not included as no recent data on opportunistic screening were available in Norway. The inclusion of this issue might have increased LYG in both invited and not‐invited groups.^53^ As breast cancer treatment data were available for the period 2006–2017, more extensive surgical treatment, lack of neoadjuvant chemotherapy and targeted treatment during the period 1996–2005 were not accounted for.^54^ This might have resulted in smaller differences in HUV losses between women with screen‐detected and symptomatic cancer and underestimation of side effects of treatment for both overdiagnosed and not overdiagnosed women. Generally, our assumptions might be viewed as overestimating the harms of mammographic screening, specifically in Model B. Further studies using similar assumptions and populations are needed to validate and improve the methodological background. However, we consider the methods used in this study relevant for screening policy assessment and modification, as our finding of positive net QALYs was robust over a wide range of assumptions.

## 5 CONCLUSION

Inviting women aged 50–69 to mammographic screening in Norway was associated with positive net QALYs ranging from 2446 to 7444 for various scenarios and 80% BC mortality transfer, when followed until age 85. The cumulative net QALYs turned positive after the first 8–16 years following the first invitation to the screening program, and benefits were sustained also after age 85.

## AUTHOR CONTRIBUTIONS

**Rick Groeneweg:** Conceptualization; investigation; writing – original draft; writing – review and editing; visualization; validation; methodology; formal analysis; data curation. **Nicolien T. van Ravesteyn:** Conceptualization; investigation; writing – original draft; methodology; validation; visualization; writing – review and editing; formal analysis; data curation; supervision. **Lindy M. Kregting:** Writing – review and editing; formal analysis; investigation; visualization; methodology; conceptualization. **Giske Ursin:** Formal analysis; visualization; investigation; methodology; writing – review and editing; conceptualization. **Solveig Hofvind:** Conceptualization; formal analysis; project administration; validation; methodology; investigation; writing – review and editing; writing – original draft. **Nataliia Moshina:** Conceptualization; investigation; writing – review and editing; validation; methodology; project administration; formal analysis; writing – original draft; visualization; data curation; supervision.

## CONFLICT OF INTEREST STATEMENT

The authors declare no conflicts of interest.

## ETHICS STATEMENT

This study has a legal basis in accordance with Articles 6(1)(e) and 9(2)(j) of the GDPR. The study was approved by the Regional Committee for Medical and Health Research Ethics (REC, south‐east D, 77226). The data were disclosed with the legal basis in the Cancer Registry Regulations section 3‐1.

## Supporting information


**Table S1.** Treatment regimens distribution for women invited to BreastScreen Norway and diagnosed with screen‐detected, symptomatic and interval cancer, 2006–2017, based on self‐reported information from responses to the questionnaire on health‐related quality of life (2).
**Table S2.** The cumulative quality‐adjusted life years (QALYs) and life years gained (LYG) for Model Microsimulation Screening Analysis (MISCAN), Model A and Model B and mortality transfer of 50%, 80% and 100% per 100,000 women aged 50–85 years invited to organized mammographic screening.
**Figure S1.** Flowchart of the study.
**Figure S2.** The relationship between the method used to model mortality reduction by breast cancer (BC) screening, and the total reduction in BC mortality between the ages 50 and 85 years.
**Figure S3.** Cumulative number of overdiagnosed cases for a group of 100,000 women, by age at diagnosis for three different levels of overdiagnosis proportion (OdP), and in the MISCAN simulation. In Models A and B, we used an OdP of 15% and 50%, respectively. As the number of overdiagnosed cases was constrained to be proportional to the number of screen‐detected cancers in each age‐group, and the incidence of breast cancer without overdiagnosis was fixed, the number of overdiagnosed cases was not linear with regard to the OdP.
**Figure S4.** Cumulative number of overdiagnosed cases per 100,000 women by age for different overdiagnosis proportions and corresponding overdiagnosis rates reported previously (1), based on all breast cancer cases including screen‐detected, interval, and symptomatic ductal carcinoma in situ or invasive breast cancer. This method of calculating the number of overdiagnosed cases was not used in the main models of the study.
**Figure S5.** Cumulative number of overdiagnosed breast cancer (BC) cases, presented as percentage (overdiagnosis proportions) of screen‐detected ductal carcinoma in situ (DCIS) and invasive breast cancer cases being overdiagnosed, by age at diagnosis for 5 different combinations of DCIS and invasive cancers. The dark blue line depicts an extreme alternative where all DCIS and all invasive breast cancer cases of TisN0M0, T1N0M0, T0N1miM0, T1N1miM0 (3) were assumed to be overdiagnosed.
**Figure S6.** Bar graph with the number of screen‐detected breast cancer (BC) cases per age from the MISCAN simulation per 100,000 women on the left y‐axis. The number of women who would have also received a BC diagnosis in the absence of screening are shown in blue. The number of women who would not have received a BC diagnosis and are thus overdiagnosed are shown in red. Overdiagnosis, as a percentage of screen‐detected BC cases, is plotted by age at diagnosis in red, using the right y‐axis. Overdiagnosis ranges from 3.6% at 52 years to 8.9% at 68 years of age.
**Figure S7.** (A–D) Number of women with breast cancer (BC) receiving various BC treatment types (hormonal therapy [HT] and chemotherapy, chemotherapy, HT, or no systemic therapy) by age at diagnosis as simulated using MISCAN for 10,000,000 women. Systemic therapy refers to HT and/or chemotherapy. (A) The number of BC cases receiving BC treatment in the presence of screening. The screen‐detected cases are shown with a dashed pattern. (B) The number of BC cases receiving BC treatment in the absence of screening. (C) The number of BC cases receiving BC treatment with the difference in treatment type between the screening and the no‐screening populations. (D) The number of overdiagnosed BC cases due to screening, and their treatment.
**Figure S8.** (A, B) Quality‐adjusted life years (QALYs) lost based on treatment harms for overdiagnosed women in relation to the treatment distribution that overdiagnosed women received. The numbers of QALYs lost due to treatment are modelled in Model A and B, their relative shapes are the same, since the harms scale linearly corresponds to the number of overdiagnosed women, but the magnitude of the harms is different. The vertical line represents the harms with the standard treatment distribution for overdiagnosed women, i.e., 28.8% for breast conserving treatment (BCT), 19.2% for mastectomy, 32% for hormonal therapy (HT) and surgery (BCT or mastectomy with or without radiation therapy), 12% for chemotherapy and surgery, and 8% for HT, chemotherapy and surgery (1). The sensitivity analysis was performed by changing the proportion of one treatment regimen, while keeping the proportions of the other treatment regimens the same, as described in the methods section.
**Figure S9.** Estimation and a sensitivity analysis of the number of prevented systemic therapies per 100,000 women. The treatment distribution for screen‐detected breast cancer (BC) was compared to the distribution in symptomatic BC. Ductal carcinoma in situ (DCIS) and invasive BC were associated with different treatment distributions, where women with DCIS tended not to receive systemic therapy (4, 5). The treatment distribution of screen‐detected cases was assumed to be equal to the treatment distribution of symptomatic cases, if there were no screening. This was a very strong assumption, because screen‐detected cancers were shown to be less aggressive (6), so the results of this figure are biased in favor of screening. The reference values for the overdiagnosis proportion were 20% for invasive BC and 50% for DCIS, and various ranges for these proportions were included in the sensitivity analyses. The prevalence of DCIS among interval cancers was used to estimate the proportion of screen‐detected DCIS that would have developed into invasive symptomatic BC. The number of screen‐detected DCIS cases that would have become invasive, was assumed to be the same as the prevalence of symptomatic DCIS in interval cancers, and the shift in the number of women received systemic therapy was calculated based the difference between the aforementioned groups. However, screening might filter out DCIS cases from the interval cancer, and hence the proportion of symptomatic DCIS in the absence of screening could be higher. Therefore, a factor of 1.2 and 1.4 times as high as the baseline situation was presented in this sensitivity analysis and was referred to as “proportion DCIS in symptomatic.” Findings of this analysis were that detecting DCIS early prevented a high number of systemic therapies, much more so than finding invasive BC early, as presented in the bottom two bars.
**Figure S10.** Total life years gained (LYG) due to screening of women aged 50–69 years and followed until 95 years of age, using 3 methods to model mortality reduction, maximum mortality reduction for ages 55–70 of 40% as in Model A, maximum mortality reduction for ages 55–70 of 20% as in Model B, and mortality reduction simulated by MISCAN. The solid lines represent scenarios with 80% mortality transfer, while the dashed lines represent scenarios with 50% mortality transfer.

## Data Availability

Research data used in the analyses can be requested from the Cancer Registry of Norway, the Norwegian Public Health Institute. Python code is publicly available on GitLab (https://gitlab.com/erasmusmc‐public‐health/early‐detection‐and‐screening/qalysbreastscreennorway). Further information is available from the corresponding author upon request.
