# Supplementary material for: Quality‐adjusted life years in the presence and absence of organized mammographic screening using data from BreastScreen Norway
Source: Int J Cancer. 2025 Dec 12;158(10):2537–48. doi: 10.1002/ijc.70272 (PMC12996758; doi:10.1002/ijc.70272)
Supplement: Supplementary file 1 — Table S1. Treatment regimens distribution for women invited to BreastScreen Norway and diagnosed with screen‐detected, symptomatic and interval cancer, 2006–2017, based on self‐reported information from responses to the questionnaire on health‐related quality of life (2). Table S2. The cumulative quality‐adjusted life years (QALYs) and life years gained (LYG) for Model Microsimulation Screening Analysis (MISCAN), Model A and Model B and mortality transfer of 50%, 80% and 100% per 100,000 women aged 50–85 years invited to organized mammographic screening. Figure S1. Flowchart of the study. Figure S2. The relationship between the method used to model mortality reduction by breast cancer (BC) screening, and the total reduction in BC mortality between the ages 50 and 85 years. Figure S3. Cumulative number of overdiagnosed cases for a group of 100,000 women, by age at diagnosis for three different levels of overdiagnosis proportion (OdP), and in the MISCAN simulation. In Models A and B, we used an OdP of 15% and 50%, respectively. As the number of overdiagnosed cases was constrained to be proportional to the number of screen‐detected cancers in each age‐group, and the incidence of breast cancer without overdiagnosis was fixed, the number of overdiagnosed cases was not linear with regard to the OdP. Figure S4. Cumulative number of overdiagnosed cases per 100,000 women by age for different overdiagnosis proportions and corresponding overdiagnosis rates reported previously (1), based on all breast cancer cases including screen‐detected, interval, and symptomatic ductal carcinoma in situ or invasive breast cancer. This method of calculating the number of overdiagnosed cases was not used in the main models of the study. Figure S5. Cumulative number of overdiagnosed breast cancer (BC) cases, presented as percentage (overdiagnosis proportions) of screen‐detected ductal carcinoma in situ (DCIS) and invasive breast cancer cases being overdiagnosed, by age at diagnosis for 5 diffe [file IJC-158-2537-s001.pdf]

Quality adjusted life years in the presence and absence of organized mammographic screening using data from BreastScreen Norway

Rick Groeneweg, Nicolien T. van Ravesteyn, Lindy M. Kregting, Giske Ursin, Solveig Hofvind, Nataliia Moshina

Table of contents

Supplementary materials and methods 2
Supplementary tables 3
Supplementary figures 5
Supplementary references 15

Supplementary materials and methods

Overdiagnosis calculation

We used two different methods to calculate the number of overdiagnosed cases in this study, and included two other methods in this additional file and the codebase. In the codebase, the function `model.py/Model.calculate_new_overdiagnoses` is the entry point to calculate numbers and proportions of overdiagnosed cases. The comments are included for explaining every step of the calculation.

Firstly, we replicated the method used in a previous study from Norway (1) to make the estimations presented in Figure A4, where we reported the *total* overdiagnosis proportion (OdP). The total OdP summarizes the proportion of overdiagnosed cases among all cases including screen-detected, interval, and symptomatic cases of both ductal carcinoma in situ (DCIS) and invasive breast cancer (BC). The total OdP was calculated from a fixed overdiagnosis rate (OdR), corresponding to the constant proportionality between the incidence before screening and the number of overdiagnosed cases; OdP = OdR / (OdR+1). The OdR was the number reported in a previous study (1), while the total OdP was much lower. The OdR of 50%, 30%, 20% and 10% corresponded to an OdP of 33%, 25%, 17% and 9%. In the code, this method was encoded as `HowToComputeOverdiagnoses.RateOfTotalIncidence`.

Secondly, the method used for Models A and B in this study, as described in the methods section, was encoded as `HowToComputeOverdiagnoses.ProportionalToScreenDetected`. Naturally, for Model MISCAN, the number of overdiagnosed cases from the MISCAN simulation was used.

Thirdly, for Figure A5, the cumulative number of overdiagnosed cases was estimated for screen-detected DCIS and invasive BC cases separately, but the method was the same as the method used for Models A and B. This was performed for five different combinations of DCIS and invasive cancers; 100% DCIS and 69.23% invasive cancers; 80% DCIS and 35% invasive cancers; 50% DCIS and 33% invasive cancers; 50% DCIS and 20% invasive cancers; and 33% DCIS and 15 % invasive cancers. In the code, this method was encoded as `HowToComputeOverdiagnoses.InvasiveAndDCISSeparately`.

Supplementary tables

Table S1. Treatment regimens distribution for women invited to BreastScreen Norway and diagnosed with screen-detected, symptomatic and interval cancer, 2006-2017, based on self-reported information from responses to the questionnaire on health-related quality of life (2)

	Screen-detected cancer N=1141	Symptomatic cancer N=642	Interval cancer N=919	Total N=2702
BCT & radiation therapy, n	343 (30.1%)	77 (12.0%)	112 (12.2%)	532 (19.7%)
Mastectomy, n	42 (3.7%)	23 (3.6%)	40 (4.4%)	105 (3.9%)
BCT/Mastectomy & hormonal therapy, n	28 (2.5%)	17 (2.6%)	25 (2.7%)	70 (2.6%)
BCT/Mastectomy & chemotherapy, n	35 (3.1%)	31 (4.8%)	49 (5.3%)	115 (4.3%)
BCT/Mastectomy, hormonal therapy & chemotherapy, n	45 (3.9%)	42 (6.5%)	48 (5.2%)	135 (5.0%)
BCT/Mastectomy, hormonal therapy, chemotherapy & radiation therapy, n	305 (26.7%)	252 (3.9%)	305 (33.2%)	862 (31.9%)
BCT/Mastectomy, hormonal therapy & radiation therapy, n	151 (13.2%)	63 (9.8%)	128 (13.9%)	342 (12.7%)
BCT/Mastectomy, chemotherapy & radiation therapy, n	177 (15.5%)	130 (20.2%)	202 (22.0%)	509 (18.8%)
Mastectomy & radiation therapy	<10*	<10*	<10*	19 (0.7%)
Only BCT	<10*	<5**	<5**	13 (0.5%)

BCT – breast conserving treatment

*A random number between 1 and 9 was used for the calculation

**A random number between 1 and 4 was used for the calculation

Table S2. The cumulative quality adjusted life years (QALYs) and life years gained (LYG) for Model Microsimulation Screening Analysis (MISCAN), Model A and Model B and mortality transfer of 50%, 80% and 100% per 100,000 women aged 50-85 years invited to organized mammographic screening

		Mortality Transfer		
		50%	80%	100%
QALYs	Model MISCAN	4087	6819	8640
	Model A	4417	7444	9462
	Model B	931	2446	3455
LYG	Model MISCAN	4553	7284	9105
	Model A	5045	8072	10,090
	Model B	2524	4038	5048

Supplementary figures

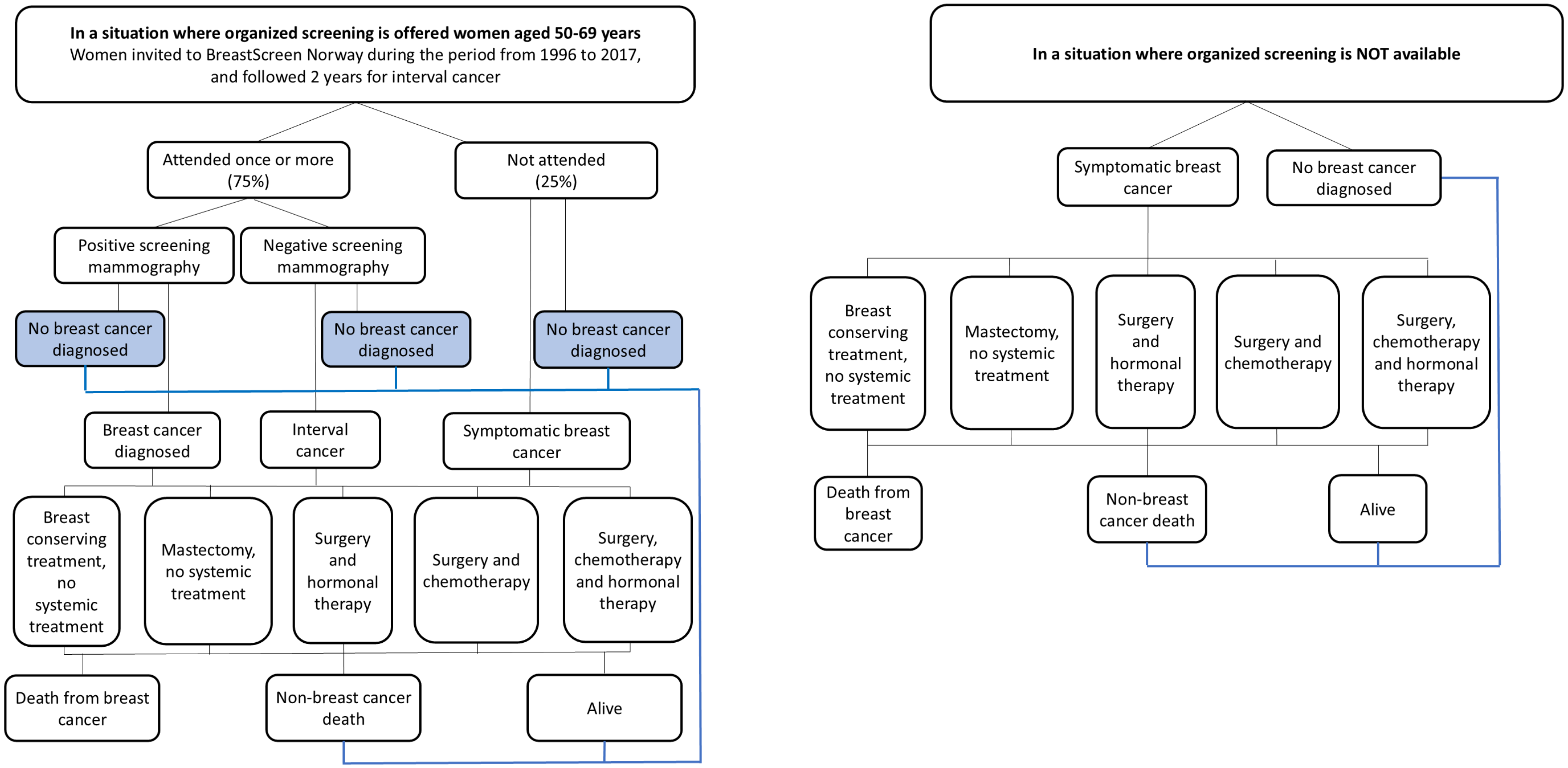

Figure S1. Flowchart of the study.

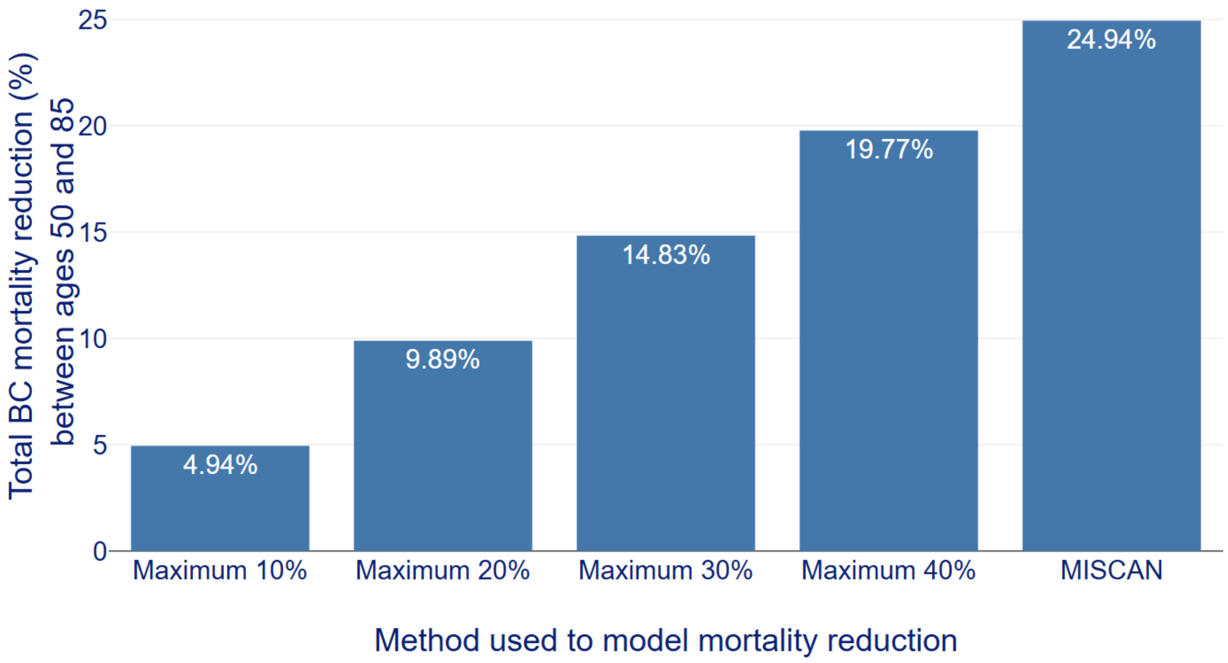

Figure S2. The relationship between the method used to model mortality reduction by breast cancer (BC) screening, and the total reduction in BC mortality between the ages 50 and 85 years.

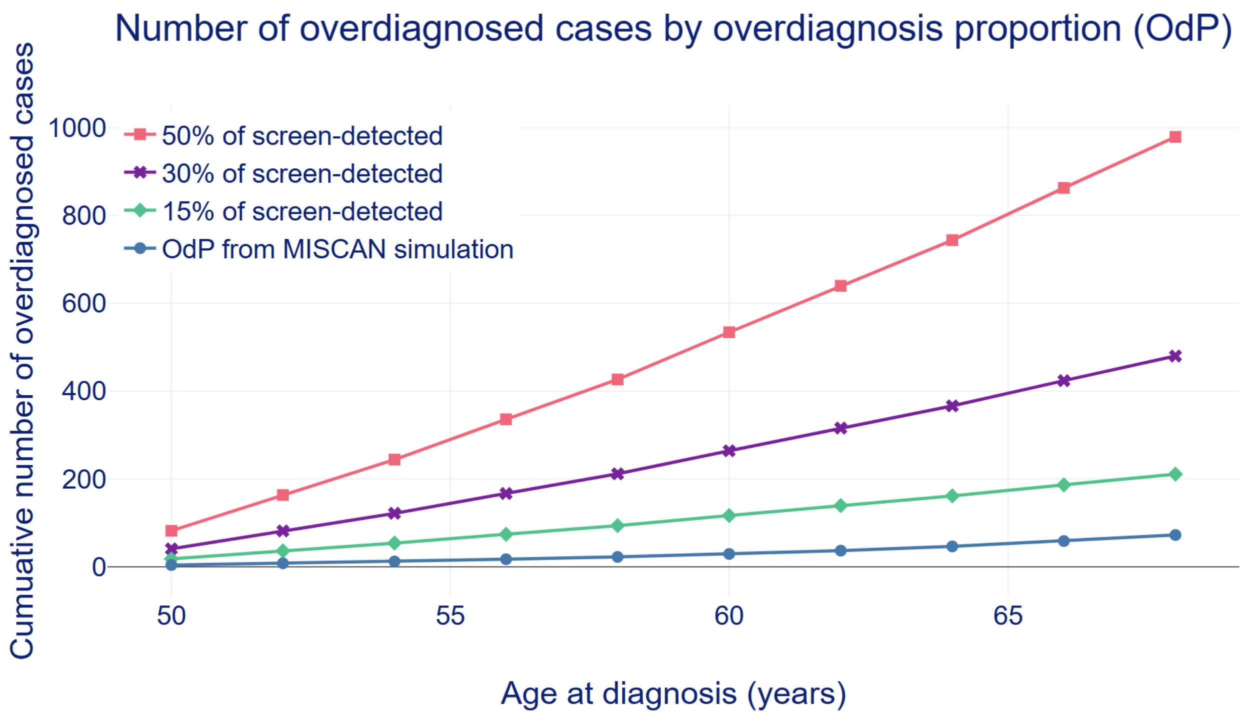

Figure S3. Cumulative number of overdiagnosed cases for a group of 100,000 women, by age at diagnosis for three different levels of overdiagnosis proportion (OdP), and in the MISCAN simulation. In Models A and B, we used an OdP of 15% and 50%, respectively. As the number of overdiagnosed cases was constrained to be proportional to the number of screen-detected cancers in each age-group, and the incidence of breast cancer without overdiagnosis was fixed, the number of overdiagnosed cases was not linear with regard to the OdP.

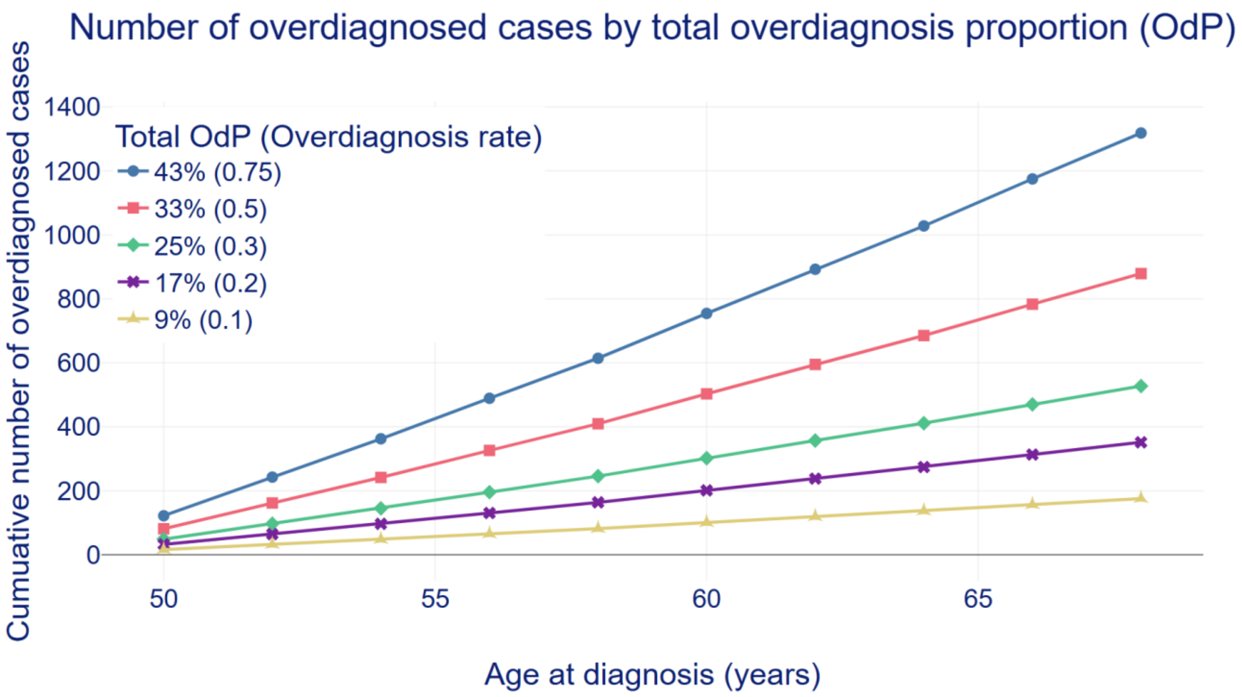

Figure S4. Cumulative number of overdiagnosed cases per 100,000 women by age for different overdiagnosis proportions and corresponding overdiagnosis rates reported previously (1), based on all breast cancer cases including screen-detected, interval, and symptomatic ductal carcinoma in situ or invasive breast cancer. This method of calculating the number of overdiagnosed cases was not used in the main models of the study.

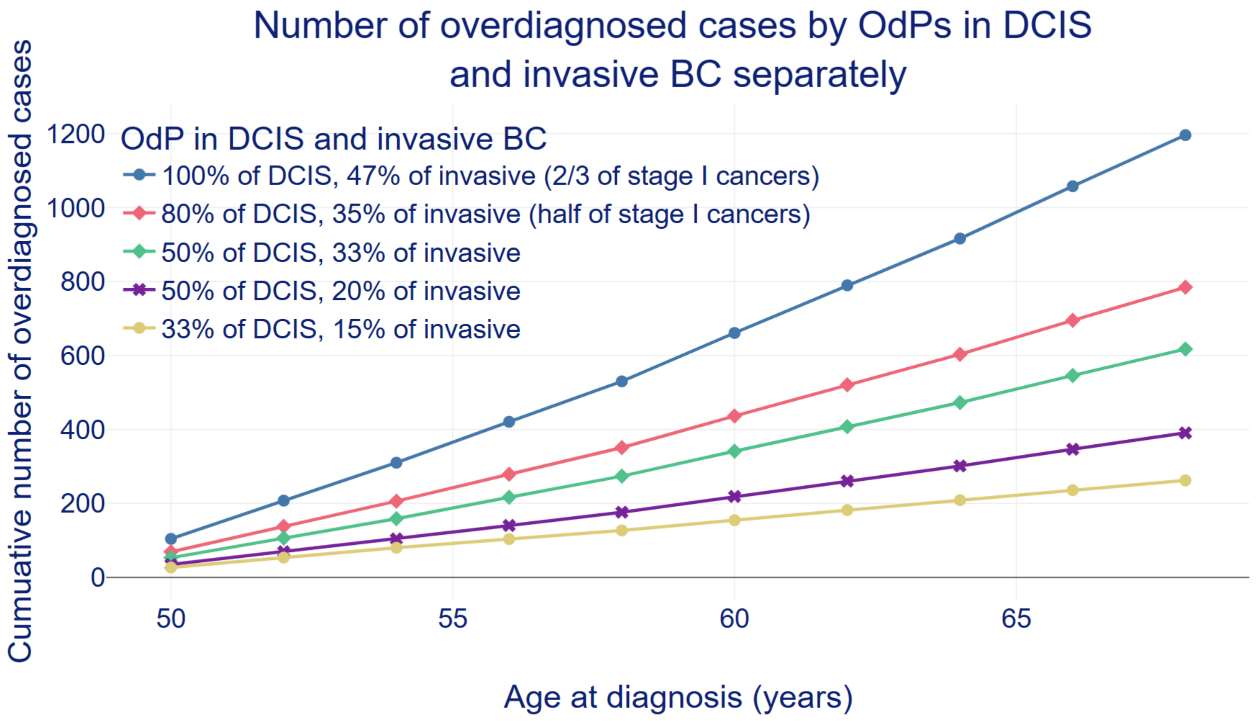

Figure S5. Cumulative number of overdiagnosed breast cancer (BC) cases, presented as percentage (overdiagnosis proportions) of screen-detected ductal carcinoma in situ (DCIS) and invasive breast cancer cases being overdiagnosed, by age at diagnosis for 5 different combinations of DCIS and invasive cancers. The dark blue line depicts an extreme alternative where all DCIS and all invasive breast cancer cases of TisN0M0, T1N0M0, T0N1miM0, T1N1miM0 (3) were assumed to be overdiagnosed.

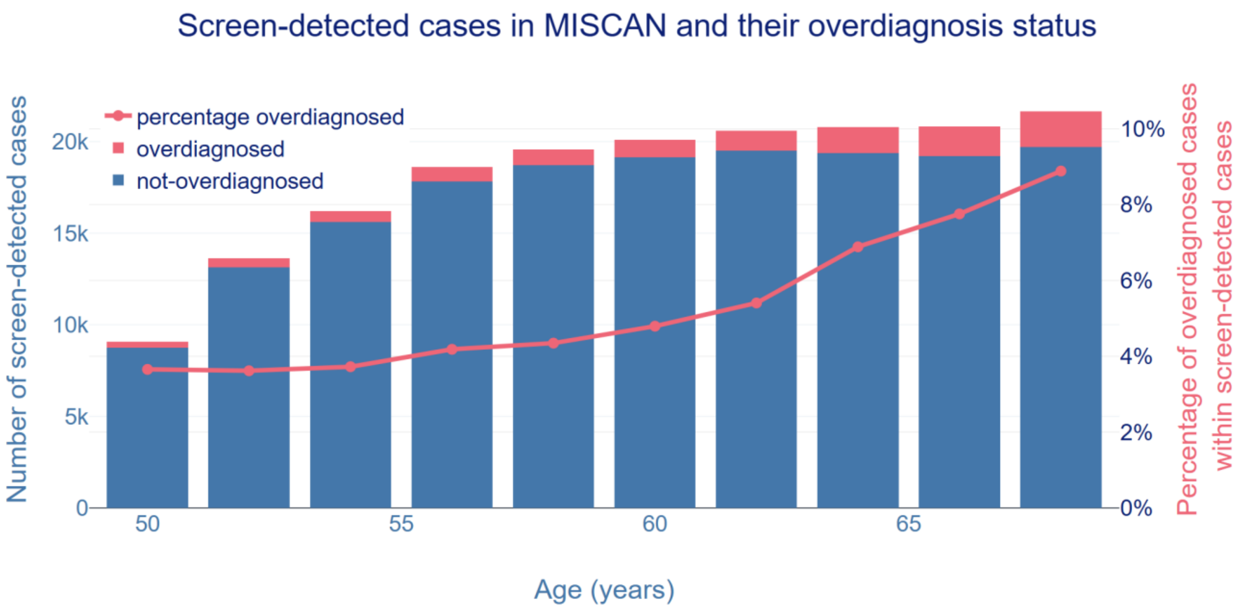

Figure S6. Bar graph with the number of screen-detected breast cancer (BC) cases per age from the MISCAN simulation per 100,000 women on the left y-axis. The number of women who would have also received a BC diagnosis in the absence of screening are shown in blue. The number of women who would not have received a BC diagnosis and are thus overdiagnosed are shown in red. Overdiagnosis, as a percentage of screen-detected BC cases, is plotted by age at diagnosis in red, using the right y-axis. Overdiagnosis ranges from 3.6% at 52 years to 8.9% at 68 years of age.

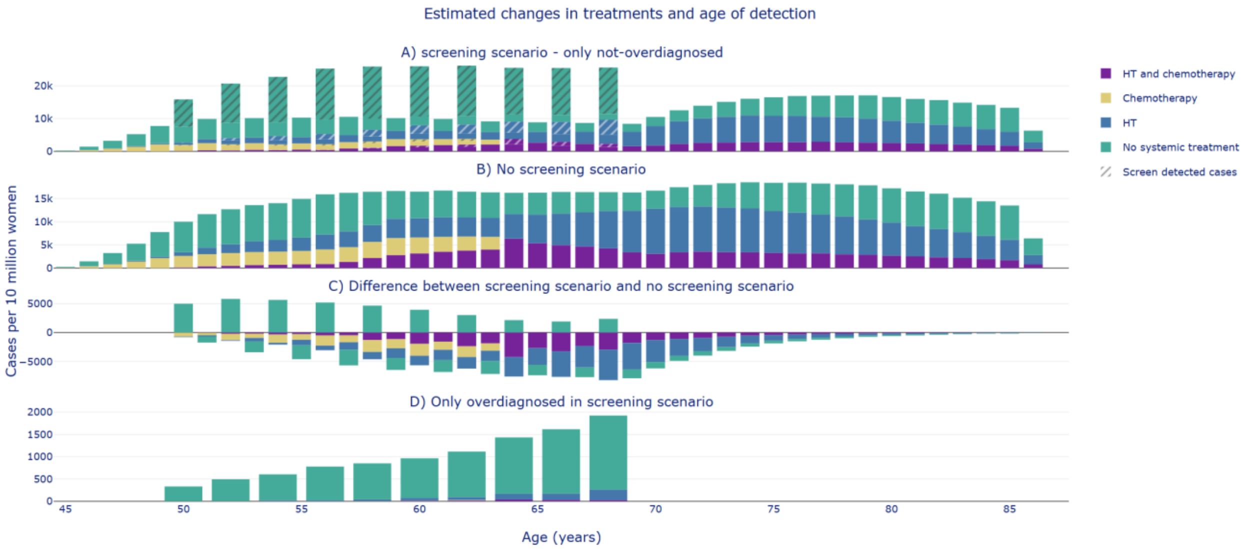

Figure S7ABCD. Number of women with breast cancer (BC) receiving various BC treatment types (hormonal therapy (HT) and chemotherapy, chemotherapy, HT, or no systemic therapy) by age at diagnosis as simulated using MISCAN for 10,000,000 women. Systemic therapy refers to HT and/or chemotherapy. A. The number of BC cases receiving BC treatment in the presence of screening. The screen-detected cases are shown with a dashed pattern. B. The number of BC cases receiving BC treatment in the absence of screening. C. The number of BC cases receiving BC treatment with the difference in treatment type between the screening and the no-screening populations. D. The number of overdiagnosed BC cases due to screening, and their treatment.

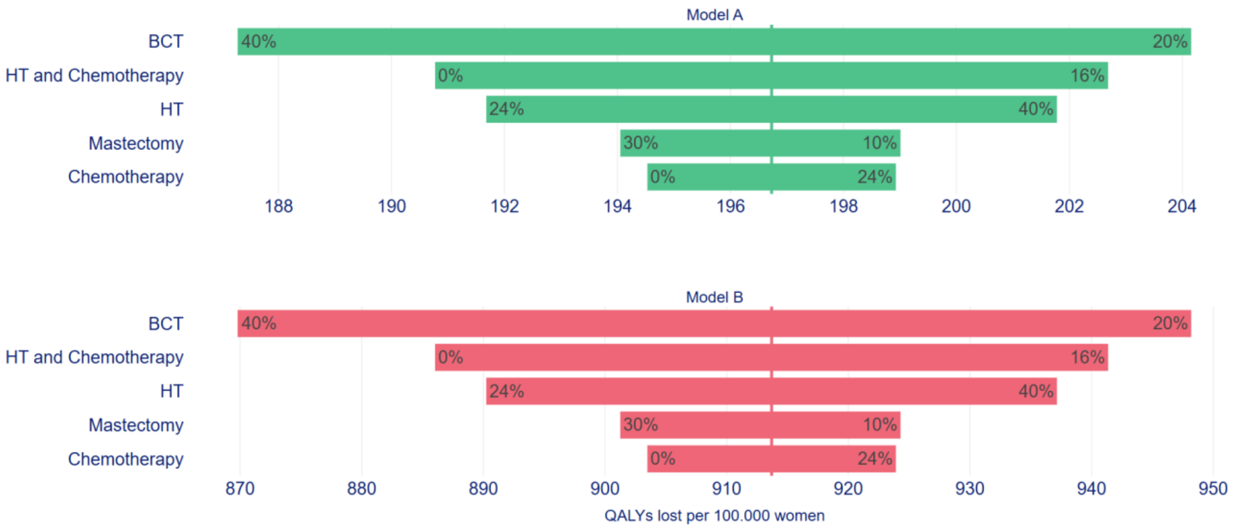

Figure S8AB. Quality adjusted life years (QALYs) lost based on treatment harms for overdiagnosed women in relation to the treatment distribution that overdiagnosed women received. The numbers of QALYs lost due to treatment are modelled in Model A and B, their relative shapes are the same, since the harms scale linearly corresponds to the number of overdiagnosed women, but the magnitude of the harms is different. The vertical line represents the harms with the standard treatment distribution for overdiagnosed women, i.e. 28.8% for breast conserving treatment (BCT), 19.2% for mastectomy, 32% for hormonal therapy (HT) and surgery (BCT or mastectomy with or without radiation therapy), 12% for chemotherapy and surgery, and 8% for HT, chemotherapy and surgery (1). The sensitivity analysis was performed by changing the proportion of one treatment regimen, while keeping the proportions of the other treatment regimens the same, as described in the methods section.

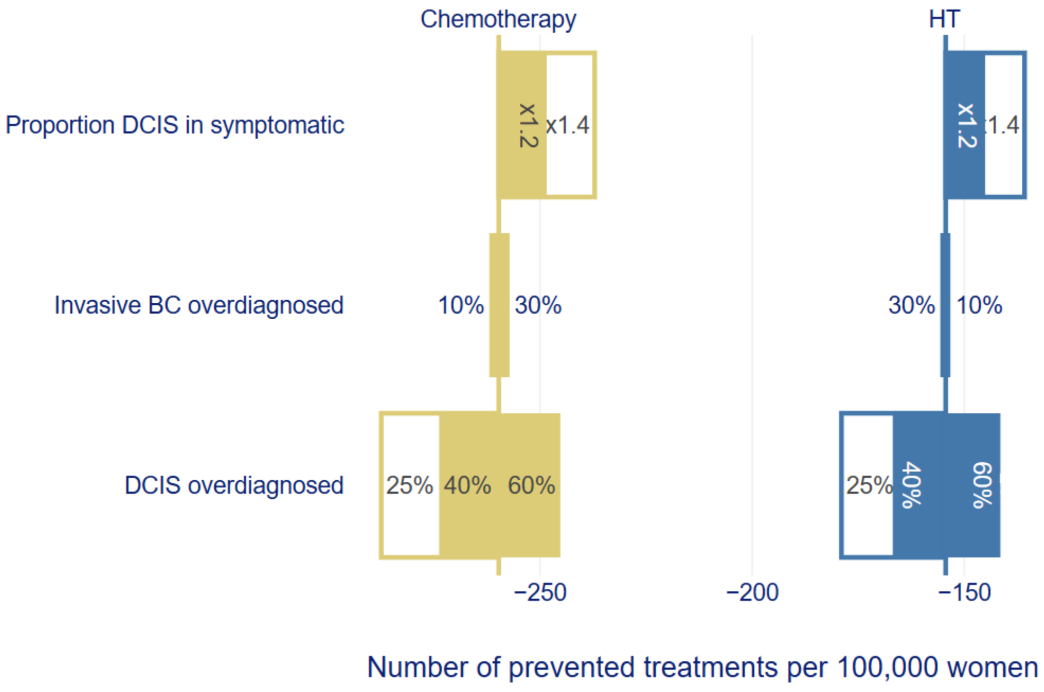

Figure S9. Estimation and a sensitivity analysis of the number of prevented systemic therapies per 100,000 women. The treatment distribution for screen-detected breast cancer (BC) was compared to the distribution in symptomatic BC. Ductal carcinoma in situ (DCIS) and invasive BC were associated with different treatment distributions, where women with DCIS tended not to receive systemic therapy (4, 5). The treatment distribution of screen-detected cases was assumed to be equal to the treatment distribution of symptomatic cases, if there were no screening. This was a very strong assumption, because screen-detected cancers were shown to be less aggressive (6), so the results of this figure are biased in favor of screening. The reference values for the overdiagnosis proportion were 20% for invasive BC and 50% for DCIS, and various ranges for these proportions were included in the sensitivity analyses. The prevalence of DCIS among interval cancers was used to estimate the proportion of screen-detected DCIS that would have developed into invasive symptomatic BC. The number of screen-detected DCIS cases that would have become invasive, was assumed to be the same as the prevalence of symptomatic DCIS in interval cancers, and the shift in the number of women received systemic therapy was calculated based the difference between the aforementioned groups. However, screening might filter out DCIS cases from the interval cancer, and hence the proportion of symptomatic DCIS in the absence of screening could be higher. Therefore, a factor of 1.2 and 1.4 times as high as the baseline situation was presented in this sensitivity analysis and was referred to as “proportion DCIS in symptomatic”. Findings of this analysis were that detecting DCIS early prevented a high number of systemic therapies, much more so than finding invasive BC early, as presented in the bottom two bars.

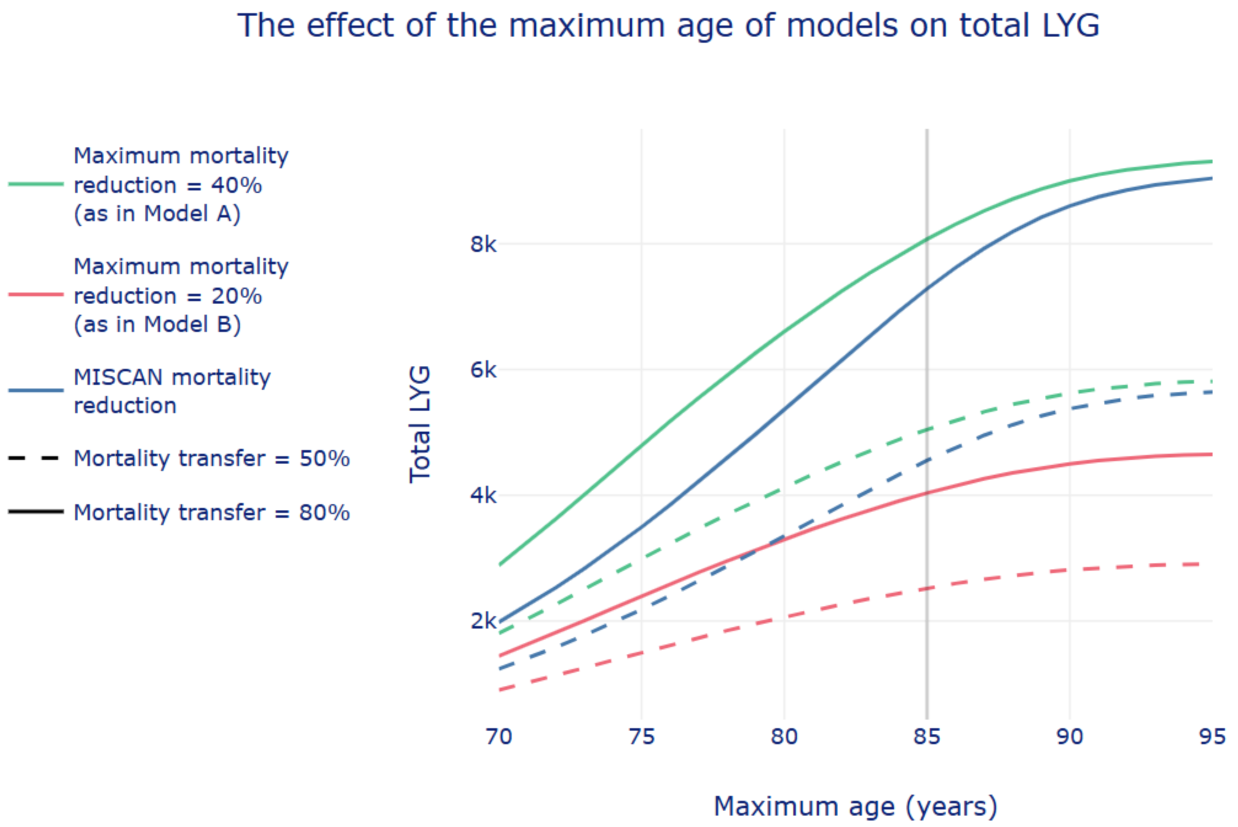

Figure S10. Total life years gained (LYG) due to screening of women aged 50-69 years and followed until 95 years of age, using 3 methods to model mortality reduction, maximum mortality reduction for ages 55-70 of 40% as in Model A, maximum mortality reduction for ages 55-70 of 20% as in Model B, and mortality reduction simulated by MISCAN. The solid lines represent scenarios with 80% mortality transfer, while the dashed lines represent scenarios with 50% mortality transfer.

Supplementary references

1. Zahl PH, Kalager M, Suhrke P, Nord E. Quality-of-life effects of screening mammography in Norway. Int J Cancer. 2020;146(8):2104-12.
2. Moshina N, Falk RS, Botteri E, Larsen M, Akslen LA, Cairns JA, et al. Quality of life among women with symptomatic, screen-detected, and interval breast cancer, and for women without breast cancer: a retrospective cross-sectional study from Norway. Qual Life Res. 2022;31(4):1057-68.
3. Hortobagyi GN, Edge SB, Giuliano A. New and Important Changes in the TNM Staging System for Breast Cancer. American Society of Clinical Oncology Educational Book. 2018(38):457-67.
4. Skjerven HK, Danielsen AS, Schlichting E, Sahlberg KK, Hofvind S. Surgical treatment of breast cancer in Norway 2003-2018. Tidsskr Nor Laegeforen. 2020;140(15).
5. Skjerven HK, Danielsen AS, Schlichting E, Sahlberg KK, Hofvind S. Treatment of Ductal Carcinoma in situ: A Register-Based Study of Norwegian Women Diagnosed between 1995 and 2018. Breast Care (Basel). 2022;17(5):486-94.
6. Domingo L, Blanch J, Servitja S, Corominas JM, Murta-Nascimento C, Rueda A, et al. Aggressiveness features and outcomes of true interval cancers: comparison between screen-detected and symptom-detected cancers. Eur J Cancer Prev. 2013;22(1):21-8.
